# Discovery of compound 1105486 as a selective inhibitor of B4GALT1: potential for pancreatic cancer therapy

**DOI:** 10.3389/fchem.2025.1651402

**Published:** 2025-08-11

**Authors:** Xu Yunyun, Mou Yiping

**Affiliations:** General Surgery, Cancer Center, Department of Gastrointestinal and Pancreatic Surgery, Zhejiang Provincial People’s Hospital (Affiliated People’s Hospital), Hangzhou Medical College, Hangzhou, China

**Keywords:** PDAC, B4GALT1, active-learning, computational-experimental integration, cytotoxicity safety window

## Abstract

Targeting aberrant β-1,4-galactosyltransferase 1 (B4GALT1) activity represents an unexplored therapeutic avenue for pancreatic ductal adenocarcinoma (PDAC). Guided by a concise active-learning structure-based workflow, we rapidly triaged 22.6 million compounds and singled out 1105486 for experimental characterization. In PANC-1 cells, the molecule suppressed proliferation with an IC_50_ of 19.8 ± 1.3 µM, while hTERT-HPNE epithelial cells retained >95% viability at concentrations up to 80 μM, indicating an encouraging initial safety window. Mechanistically, 1105486 engages the UDP-galactose pocket through stable hydrogen bonds to ARG187 and GLU313, a binding mode corroborated by 1 µs molecular-dynamics simulations and MM/GBSA energetics. Unlike previously reported glycosyltransferase inhibitors, which often lack selectivity and may affect multiple family members, 1105486 specifically targets B4GALT1 with high selectivity, occupying its unique catalytic pocket. To our knowledge, 1105486 constitutes the first reported small-molecule inhibitor of B4GALT1 and establishes a tractable chemical scaffold for optimization toward sub-micromolar potency and *in vivo* evaluation. The compound’s selective cytotoxic profile, promising physicochemical properties, and the potential for further development highlight its *in vivo* efficacy and its role as a lead candidate for the next-generation of glycosylation-directed therapeutics for PDAC.

## 1 Introduction

Pancreatic ductal adenocarcinoma (PDAC) remains one of the most lethal malignancies worldwide, accounting for substantial cancer-related mortality and exhibiting a dismal 5-year survival rate of less than 10% (Siegel et al., 2025). Despite decades of clinical research and numerous therapeutic attempts, meaningful advances in the treatment of PDAC have been limited (Halbrook et al., 2023; Hu and O’Reilly, 2024). Most approved drugs for advanced PDAC provide only modest survival benefits, and therapeutic resistance as well as toxicity to normal tissues continue to present significant obstacles (Espona-Fiedler et al., 2024; Hosein et al., 2022; Wang et al., 2023). This underscores the urgent need for novel therapeutic targets and effective, tumor-selective strategies for PDAC management.

Among the emerging molecular drivers of PDAC, β-1,4-galactosyltransferase 1 (B4GALT1) has attracted increasing attention (Sun et al., 2014; Villegas-Comonfort et al., 2012; Yuan et al., 2012). B4GALT1 is a key glycosyltransferase enzyme responsible for catalyzing the transfer of galactose to N-acetylglucosamine, a central step in the biosynthesis of glycoproteins and glycolipids (Geisler et al., 2015). Glycosylation is fundamental to a wide array of biological processes, including cell–cell recognition, immune regulation, and signal transduction (He et al., 2024; Ohtsubo and Marth, 2006). Recent cancer studies have revealed that aberrant expression or hyperactivity of B4GALT1 can reshape the glycosylation landscape of tumor cells, driving oncogenic phenotypes such as increased invasiveness, metastasis, and immune evasion (Chen et al., 2023; Cui et al., 2023). In PDAC, both proteomic and transcriptomic analyses demonstrate that B4GALT1 is frequently overexpressed in tumor tissues relative to the normal pancreas (Zhao et al., 2023). Clinically, high B4GALT1 expression is strongly associated with poor prognosis and shortened overall survival (Di Buduo et al., 2021; Ren et al., 2022). Experimental studies further confirm that silencing or knocking down B4GALT1 in PDAC cell lines leads to marked reductions in cell proliferation, migration, and invasive capacity, thus establishing its crucial role in pancreatic tumor progression (Chen et al., 2023; Cui et al., 2018; Tang et al., 2020; Wang et al., 2020).

Despite these compelling data, B4GALT1 remains largely untapped as a drug target (Tang et al., 2020). No selective, drug-like inhibitors of B4GALT1 have reached clinical development, and efforts to modulate glycosyltransferase activity have traditionally been hindered by challenges in specificity and pharmacological tractability. Most existing therapies for PDAC instead target classical oncogenic signaling (e.g., KRAS, MAPK) or metabolic pathways, often yielding unsatisfactory efficacy and off-target effects in normal tissue (Fang et al., 2023; Jiménez et al., 2024; Zhang et al., 2023). Consequently, targeting aberrant glycosylation, and specifically B4GALT1, represents a novel and rational therapeutic avenue deserving further investigation.

To address this unmet need, we applied a multidisciplinary approach that leverages advances in computational drug discovery and experimental biology (Xu et al., 2024). Utilizing homology modeling, active learning-driven virtual screening, molecular docking, and molecular dynamics (MD) simulations, we systematically explored large chemical libraries to identify novel small-molecule inhibitors targeting the B4GALT1 catalytic pocket (Zeng et al., 2024). Among the prioritized candidates, compound 1105486 emerged as a promising lead with favorable predicted binding stability and drug-like properties. Critically, we further validated its biological activity and selectivity in cell-based assays. Using the CCK8 viability assay, we observed that 1105486 inhibited the proliferation of PANC-1 pancreatic cancer cells in a dose-dependent manner (IC_50_ ≈ 20 μM), while exhibiting no significant cytotoxicity toward hTERT-HPNE normal pancreatic ductal epithelial cells—even at concentrations up to 80 μM. Statistical analysis confirmed that these effects were highly significant for cancer versus normal cells at relevant concentrations.

Together, this study provides a comprehensive workflow for the discovery and preliminary validation of selective B4GALT1 inhibitors, combining the strengths of *in silico* prediction and *in vitro* experimentation. PANC-1 cells, a widely used model for pancreatic ductal adenocarcinoma (PDAC) that exhibits high expression of B4GALT1, were chosen to evaluate the anticancer potential of compound 1105486. hTERT-HPNE cells, representing normal human pancreatic ductal epithelial cells, were included to assess the selective cytotoxicity of the compound. Our findings highlight the therapeutic relevance of B4GALT1 in PDAC, the feasibility of targeting aberrant glycosylation for anti-cancer therapy, and the translational potential of compound 1105486 as a new class of tumor-selective therapeutic agent for pancreatic cancer.

## 2 Methods

### 2.1 Homology modeling and structural optimization of B4GALT1

A three-dimensional model of B4GALT1 was constructed using the Prime module in Schrödinger Suite 2024-4. The crystal structure of human M340H-B4GALT1 in complex with GLCNAc-β1,6-Man-α-methyl (PDB ID: 4EEM) (Ramakrishnan et al., 2012) was selected as the homology modeling template. Following sequence alignment and model building, structural refinement was conducted using the Refine Protein–Ligand Complex module to optimize side-chain conformations and relieve steric clashes (Orry and Abagyan, 2012). The final model was energy-minimized using the OPLS5 force field and used in subsequent docking and simulation studies (Memar and Moosavi, 2023).

### 2.2 Compound library preparation

A total of 22,556,593 drug-like compounds were retrieved from the DrugSpaceX database (Yang et al., 2021a). Molecular preprocessing was performed using LigPrep (Schrödinger 2024-4), generating valid 3D conformers, assigning protonation states at pH 7.0 ± 0.5 using Epik, enumerating tautomers and stereoisomers, and optimizing geometries via OPLS5 energy minimization. The processed library was used for virtual screening.

### 2.3 Receptor grid generation

A receptor grid was generated using the Receptor Grid Generation tool in Schrödinger 2024-4, centered on the co-crystallized ligand site in the B4GALT1 homology model. Default van der Waals scaling parameters were used, and the inner/outer box dimensions were adjusted to accommodate diverse ligand sizes. No positional constraints were applied during grid generation.

### 2.4 Active learning-based virtual screening

Virtual screening was conducted using the Active Learning Glide workflow (Yang et al., 2021b). An initial subset of compounds was randomly selected and docked in Standard Precision (Espona-Fiedler et al.) mode using Glide (Repasky et al., 2012). A machine learning ensemble (comprising random forest and/or graph-convolutional neural network models) was then trained on extended-connectivity fingerprint descriptors (ECFP4) and the initial Glide SP scores. Predictive uncertainty was quantified using ensemble variance, enabling uncertainty sampling combined with exploitation of high-predicted-score compounds. This protocol iteratively selected molecules with either top predicted scores or highest uncertainty for redocking. A total of three active learning cycles were completed, retraining the model after each round of docking. The final top-scoring compounds were selected based on Glide SP docking scores and binding poses. This Active Learning Glide strategy has been shown to recover ∼70% of the top Glide SP hits with only ∼0.1% of the full library docked, thus greatly improving screening efficiency while maintaining hit quality.

### 2.5 Physicochemical and medicinal chemistry evaluation

To assess the developability of the screened compounds, physicochemical properties and medicinal chemistry descriptors were computed using ADMETlab 3.0 (Fu et al., 2024). Key parameters—including molecular weight (Lukauskis et al.), topological polar surface area (TPSA), octanol–water partition coefficient (LogP), number of hydrogen bond donors (HBD) and acceptors (HBA), and rotatable bonds (Hosein et al.)—were evaluated for compliance with drug-likeness filters such as Lipinski’s Rule of Five.

Additionally, compounds were analyzed for synthetic feasibility and chemical tractability using a set of medicinal chemistry filters, including the quantitative estimate of drug-likeness (QED), synthetic accessibility score (SAscore), PAINS (pan-assay interference compounds) filter, and Golden Triangle rule. Compounds satisfying multiple favorable criteria were considered promising candidates for further optimization and validation.

### 2.6 Molecular dynamics simulation in extracellular conditions

The dynamic behavior of ligand–B4GALT1 complexes was evaluated using Desmond (Schrödinger 2024-4). Each complex was solvated in a TIP3P water box with a 10 Å buffer and neutralized with 0.15 M NaCl. Systems were parameterized with the OPLS5 force field. After applying Desmond’s default relaxation protocol, 100 ns production simulations were performed under NPT conditions (300 K, 1.01 bar), using the Berendsen thermostat and barostat. Trajectory analyses included root-mean-square deviation (RMSD), root-mean-square fluctuation (RMSF), hydrogen bond persistence, and protein–ligand contact profiles, analyzed via Maestro’s Simulation Interaction Diagram tools.

### 2.7 Interaction analysis and mutagenesis validation via binding pose metadynamics

To identify key residues mediating ligand binding, protein–ligand interaction profiles were extracted from the Desmond trajectories using the Simulation Interactions Diagram. Residues involved in persistent interactions—defined as those forming contacts for over 30% of the simulation time—were considered as high-frequency interaction hotspots. These residues were individually mutated to alanine using the Residue Scanning module, and mutant complexes were structurally refined using the Protein Preparation Wizard. Binding Pose Metadynamics (BPMD), a method that enhances the exploration of binding pose stability by using metadynamics simulations to sample a broader range of ligand binding modes, was employed to assess the stability of ligand binding in both wild-type and mutant systems. Each complex underwent five independent BPMD trajectories, and CV RMSD scores were computed to evaluate pose robustness. Substantial increases in RMSD upon mutation were interpreted as evidence of interaction-driven binding stabilization (Lukauskis et al., 2022).

### 2.8 Cell viability assay (CCK-8)

The cytotoxic effects of the selected compound on human pancreatic cancer cells and control pancreatic ductal epithelial cells were evaluated using the Cell Counting Kit-8 (CCK-8; Dojindo, Japan) assay. PANC-1 (ATCC^®^ CRL-1469™) cells were obtained from the American Type Culture Collection (ATCC) and hTERT-HPNE cells were sourced from the Chinese Academy of Sciences, Type Culture Collection (CATCC). Both cell lines were seeded in 96-well plates at a density of 5 × 10^3^ cells per well and incubated overnight under standard culture conditions (37°C, 5% CO_2_) to allow cell attachment. The cells were then treated with a series of concentrations of the test compound (ranging from 0 to 80 μM) for 48 h.

Following treatment, 10 μL of CCK-8 reagent was added to each well, and the plates were incubated for an additional 2 h. Absorbance was measured at 450 nm using a microplate reader. Cell viability was calculated relative to the vehicle-treated control group, and dose–response curves were plotted using GraphPad Prism 9. The half-maximal inhibitory concentration (IC_50_) was determined by fitting a four-parameter logistic regression model. Each concentration was tested in biological triplicates, and data were expressed as mean ± standard deviation (SD).

## 3 Results

### 3.1 Structural modeling and dynamic characterization of the native B4GALT1 complex

As the available crystal structures of B4GALT1 predominantly represent the H340M mutant in complex with cofactors and substrate analogs, we constructed a homology model of the native enzyme by reverting the mutation to histidine based on the M340H-B4GALT1 structure complexed with a pentasaccharide. To evaluate the structural integrity and dynamic behavior of this reconstructed complex, an all-atom molecular dynamics (MD) simulation was performed for 500 ns.

**FIGURE 1:**
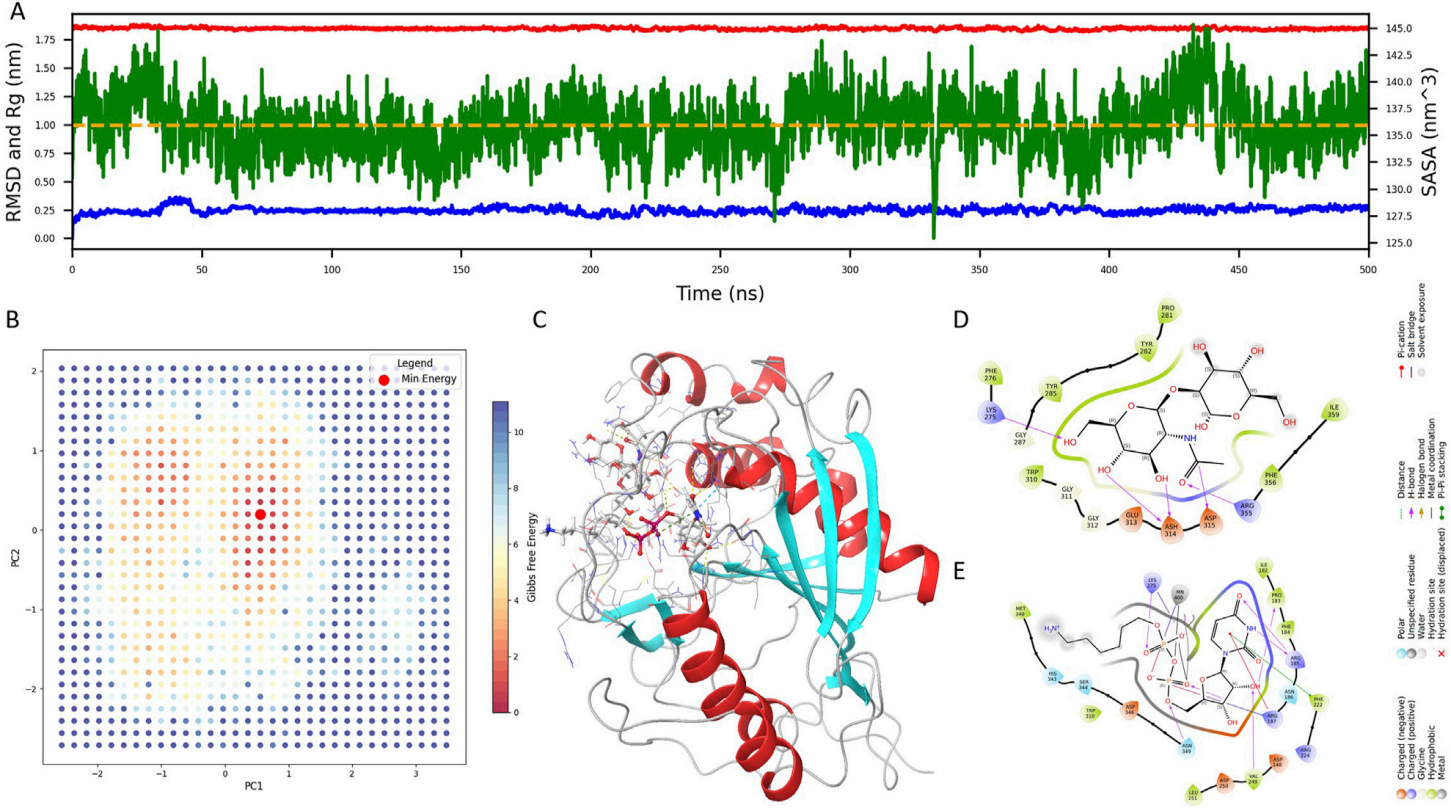
Structural modeling and molecular dynamics simulation of the native B4GALT1 complex. **(A)** Time-dependent profiles of Root Mean Square Deviation (RMSD), Solvent Accessible Surface Area (SASA), and Radius of Gyration (Rg) throughout the 500 ns molecular dynamics simulation of the native B4GALT1 complex. **(B)** Two-dimensional projection of principal component analysis (PCA) along PC1 and PC2, overlaid with the corresponding Gibbs free energy landscape. Red dots denote low-energy conformational states. **(C)** Representative minimum-energy structure extracted from the global free energy basin. **(D)** Binding interactions between B4GALT1 and the pentasaccharide ligand. **(E)** Binding interactions between B4GALT1 and the UDP-hexose donor (UDH) ligand. Key interacting residues are labeled for downstream docking constraint definition.

To monitor system equilibration, we analyzed the Root Mean Square Deviation (RMSD), Solvent Accessible Surface Area (SASA), and Radius of Gyration (Rg) over the trajectory (Figure 1A). RMSD and Rg stabilized rapidly, indicating early conformational convergence, while SASA gradually declined and reached equilibrium after approximately 350 ns, with only minor fluctuations thereafter. These metrics confirmed that the final 150 ns of the trajectory represented a well-equilibrated and stable conformation.

Principal component analysis (PCA) was subsequently applied to delineate dominant motions, and a two-dimensional Gibbs free energy landscape was generated using PC1 and PC2. As shown in Figure 1B, red dots indicate low-energy conformations, and the global minimum structure was extracted for further analysis (Figure 1C).

To enable rational inhibitor discovery, we examined the binding interface of B4GALT1 with its ligands—the pentasaccharide and UDP-hexose donor (UDH). As depicted in Figures 1D,E, the pentasaccharide forms contacts with LYS-275, ASH-314, ASP-315, and ARG-355, whereas UDH interacts with PRO-183, ARG-185, ARG-187, PHE-222, VAL-249, and LYS-275. These interaction residues will serve as spatial constraints in subsequent virtual screening efforts.

### 3.2 Active learning-driven virtual screening for B4GALT1 inhibitors

To expedite the discovery of potent B4GALT1 inhibitors, we employed an active learning-driven virtual screening strategy using the Active Learning Glide framework. During the model development phase, each training iteration involved a set of 160,000 compounds, and the process was repeated over three rounds to iteratively improve model performance. Model quality was assessed using standard regression metrics, including the coefficient of determination (R^2^), root mean square error (RMSE), and mean absolute error (MAE).

**FIGURE 2:**
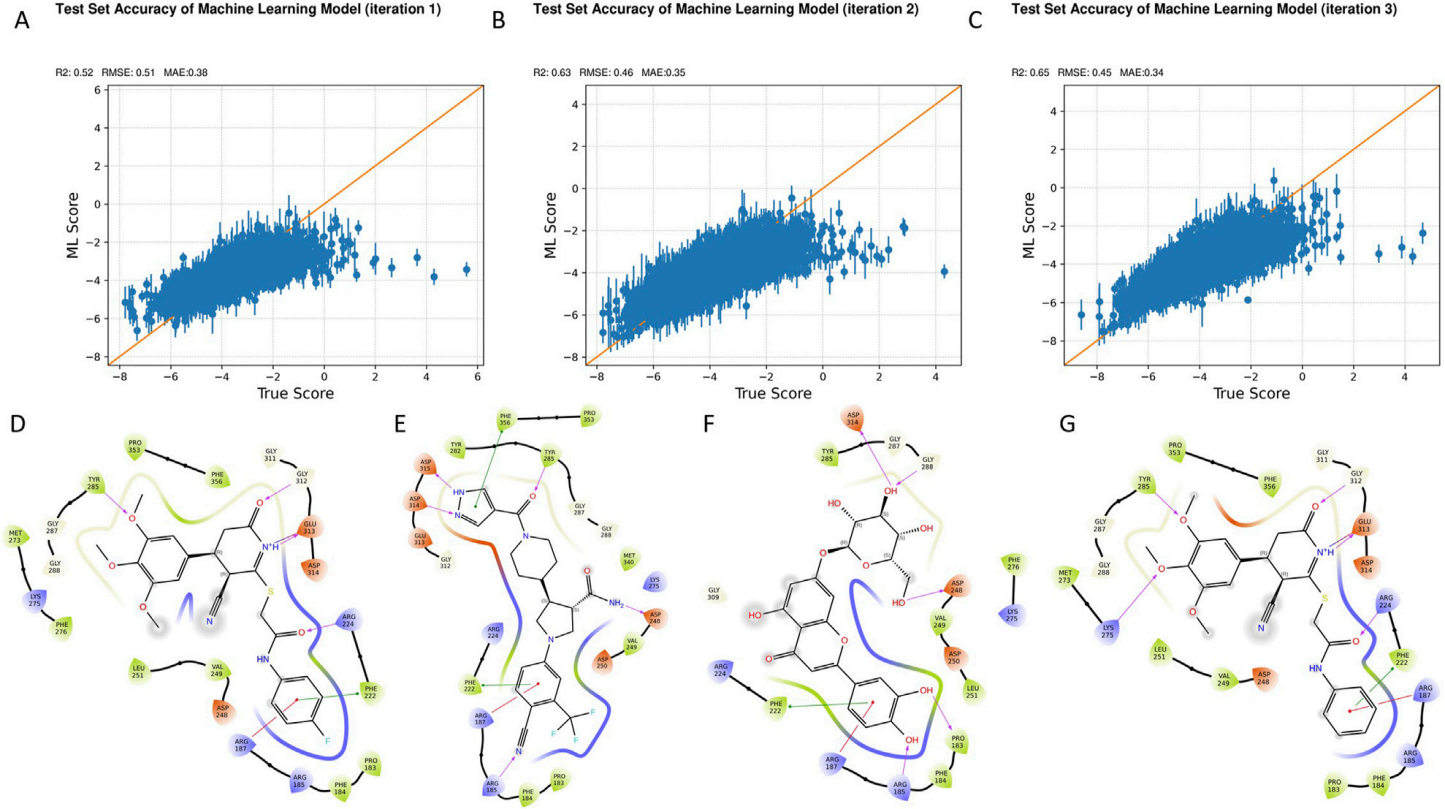
Iterative model optimization and compound prioritization through active learning-driven virtual screening. **(A–C)** Model performance metrics across three active learning iterations: R^2^ **(A)**, RMSE, and MAE **(C)**. Progressive improvements were observed with each training round. **(D-G)** Summary of the fina lfiltering process applied to the top 1.6 million compounds screened, leading to the selection of four high-confidence B4GALT1 inhibitor candidates.

As shown in Figures 2A–C, the model exhibited clear improvements over successive training rounds. The R^2^ value increased from 0.52 in Round 1 (R1) to 0.65 in Round 3 (R3), indicating enhanced correlation between predicted and experimental values. Concurrently, RMSE decreased from 0.51 to 0.45, and MAE dropped from 0.38 to 0.34, demonstrating improved predictive accuracy and reduced residual error. These trends confirm the efficacy of the active learning process in refining model performance for more reliable virtual screening.

Upon finalizing the model, a large-scale virtual screening campaign was conducted against a library of 1.6 million compounds. Post-screening filtration employed a stringent multi-parameter strategy to select drug-like compounds with favorable properties for further analysis. These criteria included Glide docking score < −7, state penalty = 0, ligand efficiency < −0.2, MMGBSA ΔG < −50 kcal/mol, and ligand strain energy < −10 kcal/mol. Additionally, compounds were evaluated for synthetic accessibility and conformance to Lipinski’s Rule of Five to ensure they were within the optimal range for drug development. Based on these combined criteria, four top-ranking compounds—1105486, 826788, 910875, and 1503232—were identified as high-confidence candidates (Figures 2D–G).

These prioritized hits will next be subjected to a comprehensive evaluation pipeline encompassing physicochemical characterization, medicinal chemistry rule-based screening, ADMET property prediction, and molecular dynamics simulations. This integrative workflow highlights the power of active learning to continuously refine predictive models and enhance hit discovery efficiency through iterative feedback and informed selection.

### 3.3 Physicochemical and medicinal chemistry evaluation of selected compounds

Following the identification of four candidate compounds via active learning-guided screening and stringent multi-parameter filtering, we next assessed their physicochemical and medicinal chemistry properties to determine their drug-likeness and suitability for downstream development.

**FIGURE 3:**
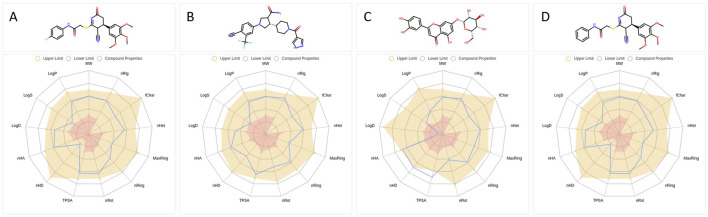
Physicochemical descriptor profiles of selected B4GALT1 inhibitor candidates. **(A–D)** Radar plots illustrating key physicochemical properties of compounds 1105486 **(A)**, 826788, 910875 **(C)**, and 1503232 **(D)**. Properties include LogP, LogD, LogS, TPSA, molecular weight, and others relevant to drug-likeness evaluation. Shaded regions represent the optimal value ranges.

Radar plots were generated to visualize key physicochemical descriptors for each compound (Figure 3). Among the four, compound 826788 exhibited a LogS value below the acceptable range, while compound 910875 showed a borderline LogD value near zero and a Topological Polar Surface Area (TPSA) that exceeded the upper threshold. Based on these findings, only compounds 1105486 and 1503232 satisfied all physicochemical criteria and were shortlisted for further evaluation.

Both candidates displayed favorable Quantitative Estimate of Drug-likeness (QED) scores, with 1503232 (0.681) marginally outperforming 1105486 (0.654), suggesting a slightly stronger overall drug-like profile. Synthetic Accessibility Scores (SAscore) were also comparable—3.326 for 1105486 and 3.275 for 1503232—indicating both compounds are synthetically tractable, with 1503232 being marginally more accessible.

The fraction of sp^3^-hybridized carbons (Fsp^3^), reflecting molecular three-dimensionality and saturation, was identical for both compounds (0.304), while Molecular Complexity Estimation (MCE-18) values were slightly higher for 1105486 (64.0) than for 1503232 (60.8), potentially offering a minor advantage in scaffold diversity. Although both compounds yielded negative Natural Product-likeness Scores (NPscore), 1503232 (−1.025) showed a greater resemblance to natural product scaffolds compared to 1105486 (−1.232), which may enhance its biological relevance.

Importantly, both candidates conformed to Lipinski’s Rule of Five, Pfizer’s Rule, and the Golden Triangle, and were devoid of any PAINS, BMS, chelating, or colloidal aggregation alerts—indicating the absence of major structural liabilities.

Furthermore, ADMET predictions for 1105486 suggest favorable pharmacokinetic properties, including good Caco-2 permeability and oral bioavailability, while also indicating minimal CYP450 inhibition and low hepatotoxicity risks. These predictions support its potential as a safe and effective therapeutic agent. However, the possibility of off-target effects, particularly with other members of the B4GALT family, and the need for further ADMET testing *in vivo* remain important considerations for future studies.

In summary, compounds 1105486 and 1503232 demonstrate complementary advantages in physicochemical stability, synthetic accessibility, and drug-likeness. These features warrant their progression into subsequent pharmacokinetic profiling and biological evaluation.

### 3.4 Molecular dynamics evaluation of binding stability and interaction profile of 1105486 with B4GALT1

**FIGURE 4:**
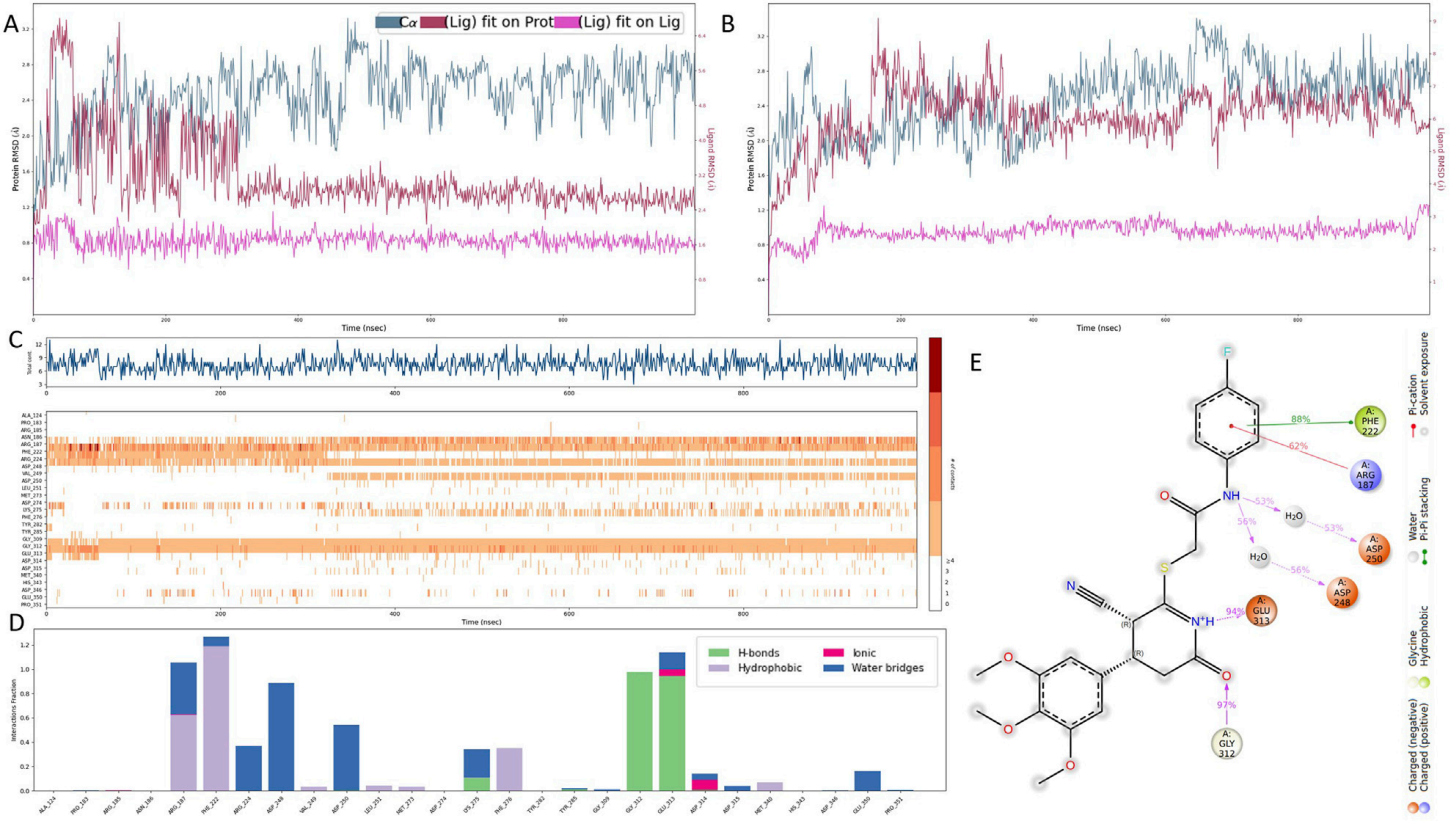
Molecular dynamics simulation of the B4GALT1–1105486 complex. **(A,B)** Ligand RMSD profiles for compounds 1105486 and 1503232 over 1 μs of simulation, indicating stability differences. **(C,D)** Time-resolved interaction analysis of 1105486 with B4GALT1, including total number of contacts and interaction type frequency. **(E)** Structural interaction model of 1105486 based on residues with interaction frequency >40% during the MD trajectory. Key residues involved in hydrogen bonds, π–π stacking, and water-bridged interactions are highlighted.

To assess the thermal stability and binding persistence of the screened compounds, 1-microsecond molecular dynamics (MD) simulations were performed for the B4GALT1–ligand complexes under room-temperature extracellular conditions. Root Mean Square Deviation (RMSD) analyses were conducted to evaluate the structural fluctuations of the ligands throughout the simulation (Figures 4A,B). Compound 1503232 exhibited a gradual increase in ligand RMSD, particularly in the later stages of the trajectory, indicating instability within the binding site. In contrast, compound 1105486 maintained a consistent and relatively low RMSD profile, suggestive of sustained binding and conformational stability. Consequently, only 1105486 was selected for subsequent interaction analysis.

Quantitative interaction profiling of 1105486 revealed a stable network of ligand–protein contacts throughout the simulation (Figures 4C,D). On average, the ligand maintained approximately five concurrent interactions, with key contributions from ARG-187, PHE-222, GLU-312, and GLY-313—residues that were previously implicated during the docking stage. These interactions predominantly involved hydrogen bonding, water-mediated contacts, and polar interactions. Notably, all four residues are located within the canonical UDP-α-D-galactose binding site of B4GALT1, suggesting that 1105486 may act as a competitive inhibitor by occupying the substrate-binding region.

To further characterize the binding interface, a high-resolution interaction model was constructed based on residue contact frequencies exceeding 40% (Figure 4E). In this model, the four core residues engaged in hydrogen bonding, π–π stacking, or salt bridge formation with 1105486. Additional interactions were observed with ASP-248 and ASP-250, which formed water bridges through interfacial solvent molecules. These auxiliary contacts likely contribute to the enhanced binding affinity and spatial stability of the ligand within the B4GALT1 active site.

These findings support the potential of 1105486 as a viable lead compound targeting B4GALT1. Future investigations will focus on structure–activity relationship optimization and evaluation of its conformational adaptability under varying physiological conditions to further validate its therapeutic potential.

### 3.5 Binding pose robustness and residue importance validated by alanine scanning and pose metadynamics

To rigorously dissect the structural and energetic roles of key binding site residues, we employed a multi-tiered validation strategy incorporating per-residue MM/GBSA energy decomposition, binding pose metadynamics (BPMD), and extended conventional molecular dynamics (cMD) simulations of alanine-substituted variants.

**FIGURE 5:**
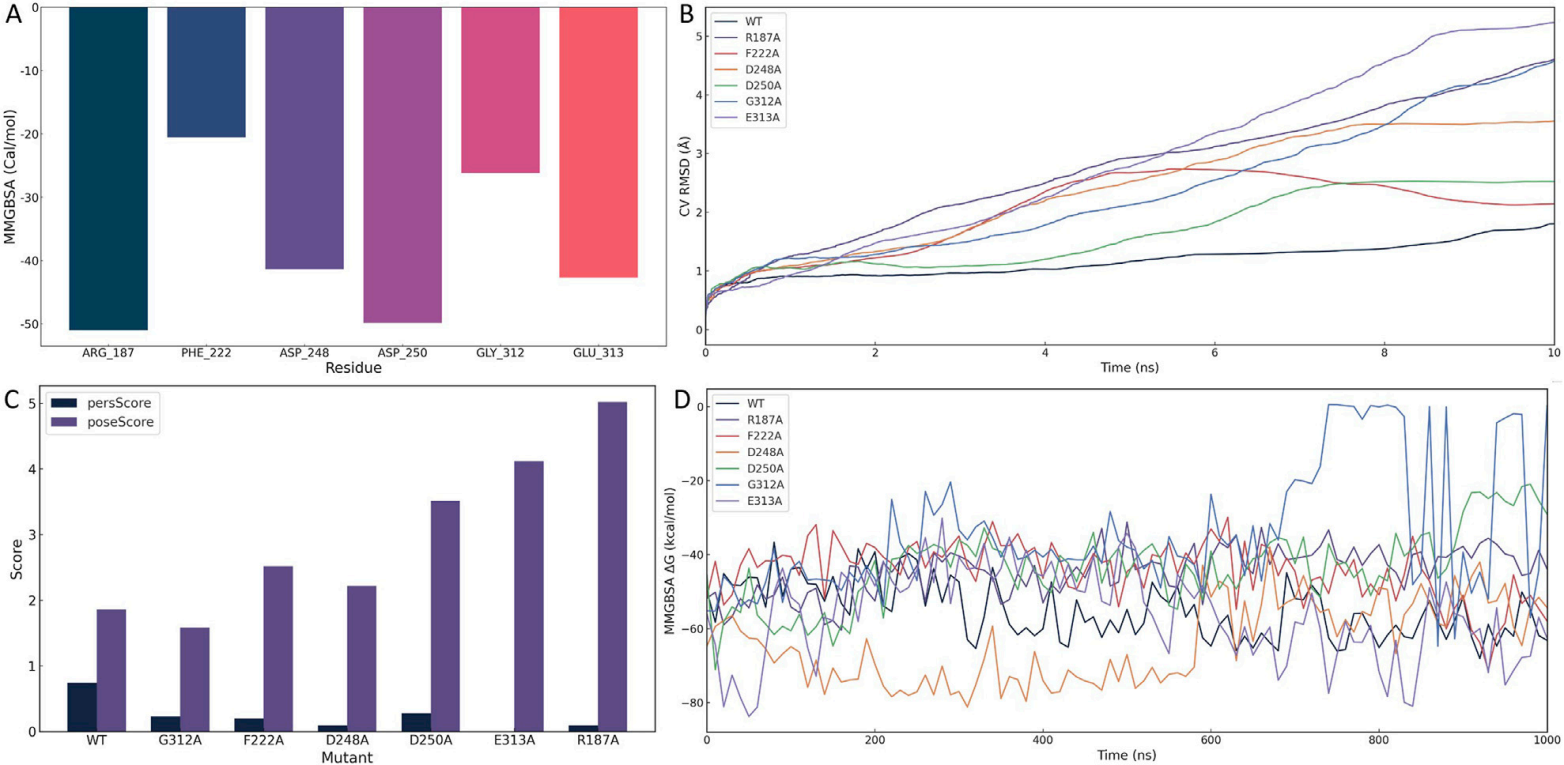
Structural and energetic validation of key residues involved in 1105486 binding via alanine scanning and binding pose metadynamics. **(A)** Per-residue MM/GBSA energy decomposition showing binding free energy contributions of six key residues. **(B)** CVRMSD trajectories of wild-type and alanine mutants during BPMD simulations, indicating the stability of the binding pose. **(C)** BPMD poseScore and persScore analysis for wild-type and mutant complexes. **(D)** Time-resolved MMGBSA ΔG values over 1 μs cMD simulations for wild-type and six mutant complexes, reflecting energetic stability.

Per-residue decomposition of MM/GBSA binding free energies identified six residues with substantial contributions to ligand stabilization: ARG-187 (−50.97 kcal/mol), ASP-250 (−49.83 kcal/mol), GLU-313 (−42.69 kcal/mol), ASP-248 (−41.38 kcal/mol), GLY-312 (−26.22 kcal/mol), and PHE-222 (−20.56 kcal/mol) (Figure 5A). These residues cluster around the uridine diphosphate–galactose (UDP-Gal) binding site, supporting the hypothesis that 1105486 mimics the natural donor substrate and competitively engages the catalytic cleft. Among these, ARG-187 and ASP-250 were the strongest energetic contributors, suggesting their primary anchoring roles.

To assess the structural impact of each residue on ligand retention, BPMD simulations were performed for the wild-type and six alanine-substituted complexes. As shown in Figure 5B, the wild-type complex maintained a low and stable collective variable RMSD (CV RMSD), whereas the R187A and E313A mutants displayed pronounced RMSD elevation (>0.6 Å), indicative of rapid conformational destabilization. Other mutants, including F222A, D248A, and G312A, showed moderate perturbations, while D250A exhibited intermediate instability.

Quantitative evaluation of pose stability using BPMD scoring further supported these trends (Figure 5C). The wild-type complex displayed a high persScore (0.74) and a low poseScore (1.86), reflecting a robust and persistent binding conformation. In contrast, the R187A and E313A mutants exhibited near-zero persScores and significantly elevated poseScores (5.018 and 4.116, respectively), confirming the collapse of the binding architecture and the critical roles of these two residues in pharmacophore anchoring.

To evaluate the energetic consequences of alanine substitution, we conducted 1 μs cMD simulations for each complex and calculated MMGBSA binding free energies at 10 ns intervals (Figure 5D). The wild-type complex consistently maintained a ΔG of approximately −55 kcal/mol. In contrast, E313A experienced an early energetic collapse, reaching −80 kcal/mol within the first 20 ns, followed by sustained instability. R187A also showed erratic energy fluctuations and periodic affinity loss, diverging from the wild-type baseline. Other mutants demonstrated moderate but consistent destabilization in ΔG, affirming their contributions to overall binding affinity.

To further characterize conformational dynamics, we conducted an additional 100 ns of cMD simulation for each mutant and analyzed three RMSD metrics: protein fit on protein RMSD, ligand fit on protein RMSD, and ligand fit on ligand RMSD (Supplementary Figure S1). Compared to the wild-type, mutant complexes exhibited increased fluctuations across all RMSD types. In particular, ligand displacement and internal distortion were evident in the R187A and E313A systems, suggesting partial or complete dissociation from the catalytic pocket.

Together, these multi-scale simulations converge on the identification of ARG-187 and GLU-313 as critical anchoring residues required for both spatial retention and energetic stabilization of 1105486. ASP-248, ASP-250, GLY-312, and PHE-222 function as auxiliary stabilizers that optimize the local electrostatic environment and shape the ligand-binding architecture.

### 3.6 Selective cytotoxicity of compound 1105486 against pancreatic cancer cells

To evaluate the selective antiproliferative effects of compound 1105486, a comparative cytotoxicity assay was conducted on normal human pancreatic ductal epithelial cells (hTERT-HPNE) and pancreatic cancer cells (PANC-1) using the CCK-8 assay.

**FIGURE 6:**
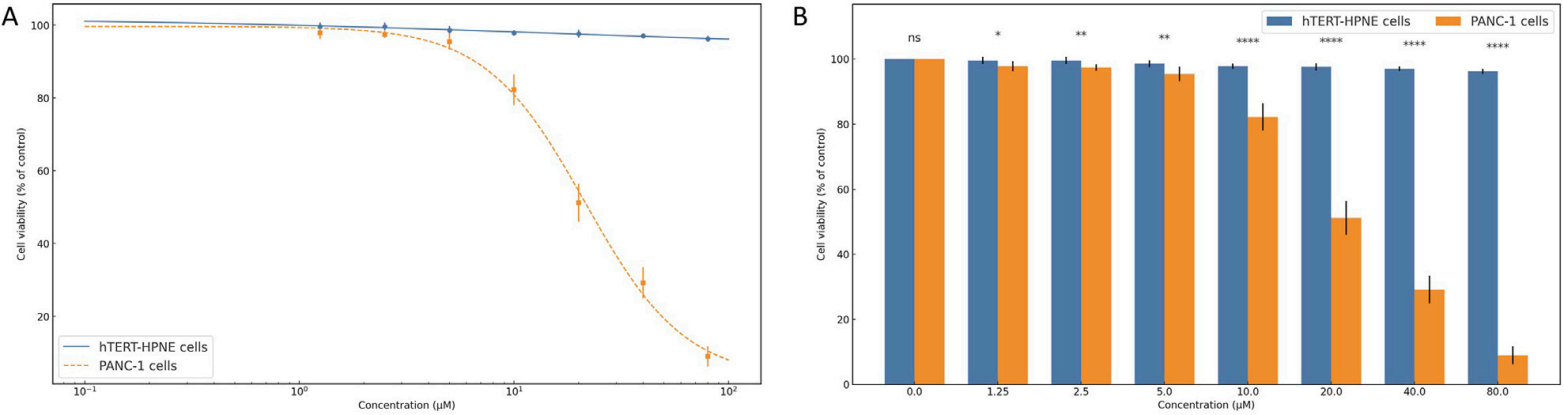
Selective cytotoxicity of compound 1105486 against pancreatic cancer cells. **(A)** Dose–response curve showing cell viability of hTERT-HPNE (gray) and PANC-1 (blue) cells following 24 h treatment with compound 1105486 (0–80 μM), as assessed by the CCK-8 assay. Data are presented as mean ± SD (n = 3). **(B)** Bar graph comparing the viability of hTERT-HPNE and PANC-1 cells at each concentration of compound 1105486. Statistical significance was determined by independent-samples t-tests. *P < 0.05; ****P < 0.0001.

As shown in Figure 6A, hTERT-HPNE cells retained high viability across all tested concentrations (0–80 μM), with average values exceeding 95% of control and minimal variation among replicates. This suggests that compound 1105486 exerts negligible cytotoxicity on non-malignant pancreatic cells, even at high doses.

In contrast, PANC-1 cells exhibited a dose-dependent reduction in viability following treatment with compound 1105486. A steep decline in cell viability was observed at concentrations ≥10 μM, with viability dropping below 10% at 80 μM. Logistic regression analysis estimated the IC_50_ value to be approximately 20 μM, highlighting the compound’s pronounced inhibitory activity against cancer cells.

Statistical comparison between the two cell lines revealed significant differences beginning at 1.25 μM, as determined by independent-samples t-tests (Figure 6B). At concentrations of 10 μM and above, the differences were highly significant (P < 0.0001), further supporting the selective cytotoxicity of compound 1105486 toward PANC-1 cells.

Collectively, these findings indicate that compound 1105486 preferentially suppresses pancreatic cancer cell viability while sparing normal epithelial cells, thereby demonstrating promising therapeutic potential with an initial safety margin.

## 4 Discussion

In this study, we identified compound 1105486 as the first selective small-molecule inhibitor of β-1,4-galactosyltransferase 1 (B4GALT1) through transcriptomics-guided target discovery and structure-based virtual screening, and validated its *in vitro* efficacy in pancreatic cancer models. Compound 1105486 demonstrated stable binding to the catalytic pocket of B4GALT1 via persistent interactions with key residues ARG-187 and GLU-312/313, showing high selectivity and competitive inhibition. Importantly, in cell-based assays, 1105486 showed dose-dependent inhibition of PANC-1 pancreatic cancer cells (IC50 ≈ 20 µM) while exhibiting negligible cytotoxicity toward normal pancreatic epithelial hTERT-HPNE cells, even at concentrations up to 80 µM. The differential viability between cancer and normal cells was statistically significant from low doses, and highly significant at concentrations ≥10 µM (P < 0.0001), confirming selective cytotoxicity. Additionally, 1105486 adheres to key drug-likeness criteria (e.g., Lipinski’s Rule of Five, Pfizer Rule) and lacks structural alerts, suggesting favorable pharmacokinetic and drug-like properties. These results position 1105486 as the first-in-class selective B4GALT1 inhibitor with promising therapeutic potential in pancreatic cancer.

This study addresses a gap in drug development targeting glycosyltransferases. B4GALT1, a key enzyme in N-glycan β-1,4 galactosylation, has been implicated in several cancers (Cui et al., 2023). In PDAC, B4GALT1 is markedly overexpressed based on transcriptomic and proteomic analyses. Clinically, high B4GALT1 expression correlates with reduced survival and increased invasiveness and metastasis. For example, elevated B4GALT1 levels have been linked to larger tumors, more lymph node metastases, and higher recurrence rates in PDAC patients (Chen et al., 2021). Functionally, B4GALT1 promotes chemoresistance in PDAC via NF-κB-mediated upregulation and stabilization of CDK11^p110 through glycosylation, leading to tumor growth and gemcitabine resistance (Ding et al., 2021). Knockdown of B4GALT1 restores chemotherapy sensitivity *in vivo*, establishing it as a critical factor in PDAC progression and therapy resistance (Zhai et al., 2025; Zhou et al., 2012). However, prior to this study, no selective small-molecule inhibitors of B4GALT1 had been reported.

Aberrant glycosylation is a hallmark of cancer, and numerous glycosyltransferases have emerged as anticancer targets (Qusairy and Rada, 2025; Thomas et al., 2021; Wang et al., 2019). Here, we compare B4GALT1 with other oncogenic glycosyltransferases. B4GALT5, another family member, catalyzes lactosylceramide synthesis and is highly expressed in multiple solid tumors (Chatterjee et al., 2023). In breast cancer, B4GALT5 stabilizes Frizzled-1 through glycosylation, activating Wnt/β-catenin signaling to maintain cancer stemness (Tang et al., 2020). In hepatocellular carcinoma (HCC), B4GALT5 overexpression correlates with reduced survival and enhances proliferation, migration, and invasion (Han et al., 2022). Mechanistically, B4GALT5 facilitates immune evasion by downregulating MHC-I via the ER-associated degradation pathway, impairing antigen presentation to CD8^+^ T cells (Xing et al., 2025). While B4GALT5 is a promising target, it currently lacks selective inhibitors. Compounds like D-PDMP inhibit glycolipid synthesis and show partial antitumor effects, but they are not selective for B4GALT5 (Hartwig and Höglinger, 2021). In contrast, our discovery of 1105486 demonstrates successful design of a highly selective inhibitor within the same family, offering a model for targeting other glycosyltransferases.

ST6GAL1, an α-2,6-sialyltransferase, is also overexpressed in several malignancies and contributes to tumor progression (Gc et al., 2022). In PDAC, prostate, breast, and ovarian cancers, ST6GAL1 enhances tumor invasiveness and worsens prognosis (Hait et al., 2022). It modifies integrins and adhesion molecules, facilitating metastasis and immune escape. For example, ST6GAL1-mediated sialylation of integrin α5 activates FAK/Paxillin signaling. Recent efforts have yielded selective ST6GAL1 inhibitors, such as SPP-037 and HZF01, which block α2,6-sialylation and suppress metastasis and angiogenesis *in vivo* (Perez et al., 2025). These examples highlight the feasibility of rational design against glycosylation enzymes. Compared to ST6GAL1, B4GALT1 lacked known inhibitors prior to this study, and our discovery of 1105486 represents a milestone in glycan-targeted therapy.

However, context-dependent effects of B4GALT1 warrant caution. In some tumors, such as HCC, reducing B4GALT1 may paradoxically enhance invasiveness via integrin-laminin pathways. This indicates that the functional role of B4GALT1 varies by tissue and microenvironment, and therapeutic inhibition must be tailored accordingly.

The cytotoxic effect of 1105486 in this study likely stems from its inhibition of key glycosylation events in cancer cells. B4GALT1 transfers galactose to N-acetylglucosamine, forming terminal galactose structures in N-glycans. Hyperactive B4GALT1 alters global glycan configurations, impacting multiple signaling pathways. Notably, B4GALT1 stabilizes CDK11^p110 and contributes to chemoresistance. It also modulates immune suppression by glycosylating PD-L1 to prevent its degradation and stabilizing the coactivator TAZ to enhance CD274 transcription. This inhibition impairs both proliferative and immunosuppressive mechanisms, making 1105486 not only cytotoxic to cancer cells but also potentially enhancing immune surveillance. In a lung adenocarcinoma model, B4GALT1 knockdown enhanced CD8^+^ T cell infiltration and sensitized tumors to anti-PD-1 therapy, suggesting that 1105486 may have additional benefits in combination with immunotherapy.

Despite its promise, this study has limitations. First, off-target effects remain untested. While modeling shows that 1105486 binds selectively to B4GALT1’s UDP-galactose pocket, the structural homology among B4GALT family members raises the possibility of cross-reactivity. Further biochemical assays are needed to confirm target specificity. Further biochemical assays are needed to confirm target specificity. Second, only two cell lines were used. Given PDAC heterogeneity, additional models (e.g., 3D organoids, PDX) are needed to validate efficacy. Moreover, we recognize that the biological evaluation of compound 1105486 was limited to only two models—PANC-1 (a classical PDAC cell line) and hTERT-HPNE (normal pancreatic epithelial cells)—which does not fully capture the high phenotypic and genetic heterogeneity of pancreatic ductal adenocarcinoma (PDAC). Established PDAC cell lines such as AsPC-1 and MIA PaCa-2 harbor distinct driver mutations, metabolic profiles, and drug sensitivity patterns; for example, MIA PaCa-2 exhibits unique genomic and metabolic features compared to PANC-1, reflecting their roles in modeling tumor heterogeneity *in vitro*. Additionally, patient-derived organoids (PDOs) faithfully recapitulate primary tumor architecture and molecular characteristics and offer high predictive value for drug response. Unfortunately, expansion into these additional models was beyond the scope of the present study due to resource and time constraints. To address this, future work will assess 1105486 across a broader panel of PDAC systems—including AsPC-1, MIA PaCa-2, BxPC-3 cell lines, patient-derived organoids, and PDX models—to robustly evaluate efficacy, selectivity, and translational potential across clinically relevant contexts. Third, *in vivo* activity and pharmacokinetics are unknown. Although 1105486 shows favorable predicted properties, animal studies are needed to confirm bioavailability, metabolism, and safety. Long-term B4GALT1 inhibition may have systemic effects, and toxicity in hematologic or neural systems must be monitored.

Given B4GALT1’s role in various cancers and its potential as a target for therapy, 1105486 serves as the first-in-class selective small-molecule inhibitor in this pathway. The potential to combine this inhibitor with existing therapies, including chemotherapy or immunotherapy, may further enhance its therapeutic benefit and mitigate the limitations observed with conventional treatment strategies.

Nevertheless, this glycosylation-targeted strategy offers several advantages. Unlike kinase or growth factor inhibitors, glycosyltransferase inhibitors like 1105486 bypass conventional resistance mechanisms. Glycosylation is more tumor-specific, offering a broader therapeutic window. The low toxicity of 1105486 toward normal cells supports this. Moreover, these inhibitors may synergize with immunotherapy. As B4GALT1 promotes PD-L1 stability, its inhibition could enhance PD-1/PD-L1 blockade. Combination with chemotherapy or radiotherapy may also be effective, as glycosylation modulates DNA damage response and apoptosis.

Future studies should assess the *in vivo* efficacy of 1105486 in PDAC mouse models, including effects on metastasis and survival. Medicinal chemistry optimization could improve potency and reduce dosing. Resistance mechanisms should be explored by profiling glycan or transcriptomic changes upon long-term exposure. If preclinical data are favorable, clinical development may be pursued. Given B4GALT1’s role in leukemia and lung cancer, 1105486 or its analogs may have broader applications. Recent preclinical work supports combining B4GALT1 inhibition with immunotherapy: in a lung adenocarcinoma model, genetic or pharmacological inhibition of B4GALT1 enhanced CD8^+^ T-cell infiltration and synergized with anti–PD-1 therapy. Based on this, we plan to evaluate 1105486 in orthotopic PDAC mouse models to assess monotherapy efficacy and PK/PD profiles, tissue distribution, and tolerability. Additionally, we will test combination regimens with gemcitabine—given B4GALT1’s role in chemoresistance—and with checkpoint blockade (anti–PD-1/PD-L1), to explore potential synergy in overcoming the immunosuppressive tumor microenvironment typical of PDAC. These planned studies aim to establish preclinical proof-of-concept for therapeutic combinations and inform eventual translation of 1105486 toward clinical trials.

Overall, this work exemplifies a multidisciplinary approach to targeting glycosylation in cancer. By identifying a potent and selective B4GALT1 inhibitor, we lay the foundation for a new class of anticancer agents. As our understanding of tumor glycomics deepens, glycosyltransferase inhibitors like 1105486 may enter the clinical arena, expanding precision oncology options for patients with difficult-to-treat malignancies such as pancreatic cancer.

## Data Availability

The raw data supporting the conclusions of this article will be made available by the authors, without undue reservation.
