# Supplementary figures and images for: Discovery of compound 1105486 as a selective inhibitor of B4GALT1: potential for pancreatic cancer therapy
Source: Front Chem. 2025 Aug 11;13:1651402. doi: 10.3389/fchem.2025.1651402 (PMC12375551; doi:10.3389/fchem.2025.1651402)

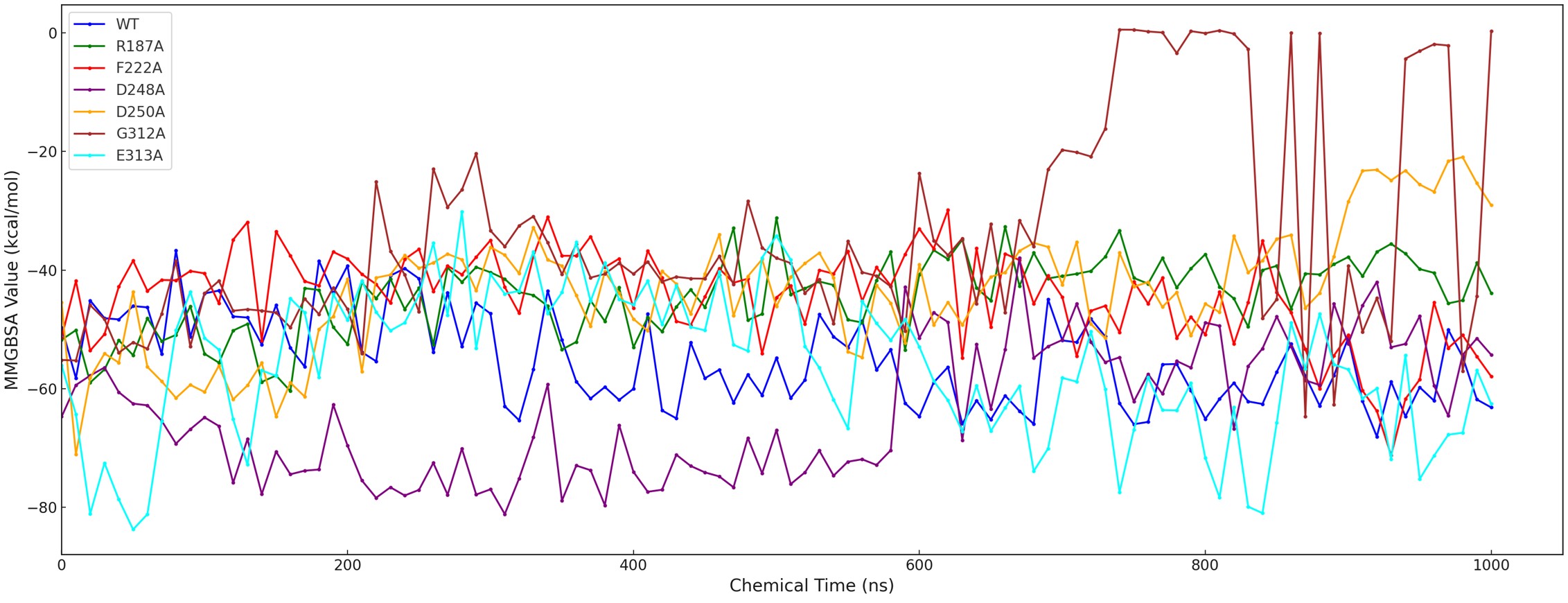

Supplement: Supplementary file 1 [file Image1.jpeg]
